# Surgical conditions in experimental laparoscopy: effects of pressure, neuromuscular blockade, and pre-stretching on workspace volume

**DOI:** 10.1007/s00464-024-11338-0

**Published:** 2024-10-24

**Authors:** F. Sterke, W. van Weteringen, P. A. van der Zee, J. van Rosmalen, R. M. H. Wijnen, J. Vlot

**Affiliations:** 1 Department of Pediatric Surgery, Erasmus MC Sophia Children’s Hospital, University Medical Center Rotterdam, PO box 2040, 3000 CB Rotterdam, The Netherlands; 2 Department of Biomechanical Engineering, Delft University of Technology, Delft, The Netherlands; 3 Department of Anesthesiology, Erasmus MC, University Medical Center Rotterdam, Rotterdam, The Netherlands; 4 Department of Biostatistics, Erasmus MC, University Medical Center Rotterdam, Rotterdam, The Netherlands; 5 Department of Epidemiology, Erasmus MC, University Medical Center Rotterdam, Rotterdam, The Netherlands

**Keywords:** Surgical workspace, Insufflation, Neuromuscular blockade

## Abstract

**Background:**

Establishing a pneumoperitoneum for laparoscopy is common surgical practice, with the goal to create an optimal surgical workspace within the abdominal cavity while minimizing insufflation pressure. Individualized strategies, based on neuromuscular blockade (NMB), pre-stretching routines, and personalized intra-abdominal pressure (IAP) to enhance surgical conditions are strategies to improve surgical workspace. However, the specific impact of each factor remains uncertain. This study explores the effects and side-effects of modifying intra-abdominal volume (IAV) through moderate and complete NMB in a porcine laparoscopy model.

**Methods:**

Thirty female Landrace pigs were randomly assigned to groups with complete NMB, regular NMB and a control group. Varying IAP levels were applied, and IAV was measured using CT scans. The study evaluated the maximum attainable IAV (*V*_max_), the pressure at which the cavity opens (p_0_), and the ease of expansion (*λ*_exp_). Cardiorespiratory parameters, including peak inspiratory pressure (PIP), mean arterial pressure (MAP), heart rate (HR), and cardiac output (CO), were continuously recorded to evaluate side-effects.

**Results:**

There were no significant weight differences between NMB groups (median 21.1 kg). Observed volumes ranged from 0 to 4.7 L, with a mean *V*_max_ of 3.82 L, mean* p*_0_ of 1.23 mmHg, and mean *λ*_exp_ of 0.13 hPa^−1^. NMB depth did not significantly affect these parameters. HR was significantly increased in the complete NMB group, while PIP, MAP, and CO remained unaffected. Repeated insufflation positively impacted* V*_max_; ease of opening; and expanding the cavity.

**Conclusion:**

In this porcine model, the depth of NMB does not alter abdominal mechanics or increase the surgical workspace. Cardiorespiratory changes are more related to insufflation pressure and frequency rather than NMB depth. Future studies should compensate for the positive effect of repeated insufflation on abdominal mechanics and surgical conditions.

**Supplementary Information:**

The online version contains supplementary material available at 10.1007/s00464-024-11338-0.

When establishing a pneumoperitoneum for laparoscopy, there is a limit to expanding the surgical workspace and the abdominal cavity. The goal is to create an adequate workspace at the lowest possible intra-abdominal pressure [1], which is determined by factors including patient anatomy, obesity, prior surgery, instrument design, surgeon experience, and patient positioning [2]. The abdominal compliance is unique for every patient. Díaz-Cambronero et al. showed that patients undergoing colorectal laparoscopic surgery benefit from a strategy consisting of deep (or complete) neuromuscular blockade (NMB), pre-stretching, and the lowest possible intra-abdominal pressure (IAP) [3]. However, the individual contribution of these factors to surgical workspace remains unknown.

Numerous studies have investigated the potential benefit of complete over moderate NMB [4–13]. Other studies have focused on investigating low IAP in combination with complete neuromuscular block, with varying conclusions [14, 15]. All of these studies use surgical rating scales that have low sensitivity for detecting changes in the size of the surgical workspace, making it difficult to generalize the results to other patient populations [16].

Developing generalizable models could help differentiate between the impacts of the individual facets of the proposed individualization strategy. Currently, only two generalizable models exist [17, 18]. Unfortunately, these models do not include predicting the effect of NMB or pre-stretching. Data acquired using volumetric imaging techniques, combined with the effects of NMB and pre-stretching, could be a first step toward investigating the impact of independent strategies and providing more insights into abdominal mechanics.

This study used volumetric computed tomography analysis to quantify surgical workspace during moderate and complete NMB. The study was conducted in a porcine model for laparoscopy. The hypothesis anticipated a significant difference in intra-abdominal volume (IAV). The effect of NMB was investigated using a protocol in which multiple insufflation runs were performed while mechanical ventilation pressures and hemodynamic parameters were continuously logged [19].

## Materials and methods

### Design

The effects of NMB and pre-stretching were investigated in an established porcine model for abdominal insufflation [20]. Before each of the 30 experiments, subjects were randomized to three groups: complete NMB, moderate NMB, or no NMB (control). The sample size of ten animals per group was determined by establishing a detection limit of 20% or 500 mL gain in abdominal workspace volume. This calculation was based on a two-sided *t* test with a significance level of 0.05 and a statistical power of 80%, assuming a normal distribution. Initially considering a group size of 9, it was subsequently adjusted to 10 per group to safeguard against the potential loss of statistical significance resulting from unforeseen events, such as the death of an animal during the study.

In each animal, the abdomen was insufflated three times: initial insufflation and two repetitions. During each repeated insufflation, the insufflation pressure was increased stepwise to create detailed compliance curves. The IAP was sustained at 0, 3.75, 6, 7.5, 9, 10.5, 12, 13.5, and 15 mmHg, respectively. This range included 0 mmHg to acquire a baseline, and 3.75 mmHg to investigate what happens when the insufflation pressure is equal to the PEEP set on the mechanical ventilator (3.75 mmHg = 5 cmH_2_O). The other values were chosen to systematically cover the range of insufflation pressures used in clinical practice. At every step, the insufflation pressure was sustained for 3 min to allow the subject time to adjust to the new insufflation pressure. After this accommodation period, a CT scan was obtained during an end-expiratory breath-hold.

Rocuronium was titrated according to the NMB level defined by randomization. The level of NMB was maintained based on both train of four (TOF) and post-tetanic count (PTC) measurements. TOF and PTC were measured by stimulating the adductor muscles of the lower extremities. Post-tetanic depletion of the neuromuscular junction was avoided by measuring TOF and PTC on two different extremities. Rocuronium was infused at the jugular and femoral veins.

Simultaneous infusion at both veins was preferred because pilot experiments showed a decreased NMB measured at the lower extremities during abdominal insufflation, likely due to compression of the lower vasculature and subsequently decreased perfusion.

The following levels of NMB were used according to the definition described by Biro et al. [21]:

1. Complete NMB: TOF = 0/4 PTC < 1/10
2. Moderate NMB: TOF = 1/4–3/4 and PTC = 10/10
3. Control without NMB: TOF = 4/4 and PTC = 10/10

The level of NMB was verified after every CT scan using TOF. For every two CT scans, the PTC level was verified. If needed, the infusion rate of rocuronium was adjusted to keep the level of NMB within the predefined range.

### Subjects

Measurements were performed in a 20-kg female Landrace porcine model. The animals were obtained from a specific pathogen-free farm. An enriched environment was provided and, if logistics allowed, animals were kept in groups. During a one-week accommodation period, the institute’s animal facility provided care and animals had free access to water and food. On the day of the experiment, the animals only had access to water. Animals were excluded if the cardiorespiratory physiology was affected by anatomic abnormalities. This study was registered at the Dutch Central Authority for Scientific Procedures on Animals and registered under license number AVD101002015180. Institutional approval was given by the Animal Welfare Body of Erasmus MC, University Medical Center Rotterdam, protocol number 15-180-02.

### Preparations

Initial sedation was provided via intramuscular injection with midazolam (40 mg/kg), ketamine (1 mg/kg), and atropine (0.03 mg/kg). Fifteen minutes were given to ensure the onset of sedation. Instrumentation started by cannulation of the marginal ear vein (20 gauge). For mechanical ventilation, the animal was intubated using an endotracheal tube (size ~ 7 mm), and lidocaine spray was used to suppress the cough reflex. The animal was weighed before placement in a supine position onto the measurement CT slide. A mechanical ventilator (Fabian HFO, ACUTRONIC Medical Systems AG, Hirzel, Switzerland) was connected and set to volume guarantee mode with the tidal volume set to 7.5–8.0 mL/kg. For maintenance of anesthesia during instrumentation, propofol and sufentanyl were provided through the cannulated ear vein.

To ensure repeatable measurement conditions, mechanical ventilation was managed using an automated system throughout the measurement protocol [22]. The automated system was compatible with tidal volume guarantee mode. Oxygenation was maintained by adapting the fraction of inspired oxygen. The carbon dioxide levels were managed by altering the respiratory rate to target an end-tidal pCO_2_ of 7.0 kPa (52.5 mmHg).

Arterial and central venous lines were placed for hemodynamic monitoring. After this, both NMB monitors were placed on both lower extremities (Dräger TOFScan, Drägerwerk AG & Co. KGaA, Lübeck, Germany).

A 10-mm trocar (VersaOne™, Medtronic, Minneapolis, USA) was placed at the subumbilical midline. After insertion, intraperitoneal placement was verified endoscopically. For creating the pneumoperitoneum, a commercially available CO_2_ insufflator was used (Endoflator 40, Karl Storz SE & Co. KG, Tüttlingen, Germany) with an inline custom-built device for high-frequency measurement and additional pressure control.

### Measurements

#### Intra-abdominal volume

CT scans were obtained using a Somatom Force scanner (Siemens Healthcare GmbH, Erlangen, Germany) and reconstructed with a 1 mm slice thickness. The IAV was measured using Myrian imaging software (Version 2.6.5 Research Edition, Intrasense, Montpellier, France). For this, the surgical workspace was automatically segmented, visually checked, and manually corrected.

#### Respiratory pressures

To analyze changes in respiratory mechanics, PEEP and tidal volume were kept constant such that the peak inspiratory pressure (PIP) reflects changes in respiratory compliance. The hemodynamic response was evaluated based on heart rate, blood pressure, and cardiac output. These were monitored using a hemodynamic patient monitor (PulsioFlex monitor, Getinge AB, Göteborg, Sweden). Samples from the mechanical ventilator and hemodynamic patient monitor were recorded at a one-second interval. The sample at 5 s before the end-expiratory breath-hold needed for the CT scan was used for further analysis.

#### Arterial pressure, heart rate, and cardiac output

The effects of NMB, repetition, and the insufflation step on the circulatory system were investigated using mean arterial blood pressure (MAP), measuring heart rate (HR), and cardiac output (CO). These parameters were sampled simultaneous to the respiratory pressures.

### Analysis

#### Abdominal mechanics

**Fig. 1:**
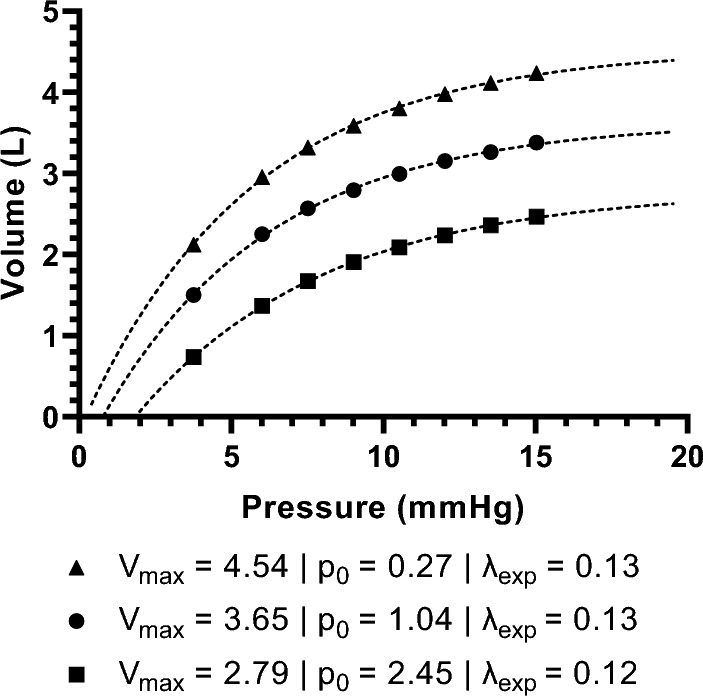
Abdominal pressure–volume curves in three different subjects (▲, ●, and ■) and estimated parameters. The corresponding models (–) are extrapolated to provide a visual explanation. *V*_max_ relates to the horizontal asymptote and* p*_0_ relates to the location on the horizontal axis when the volume equals zero. The curvature is described by *λ*_exp_

An existing model for the evaluation of abdominal compliance was used for the analysis [23]. This model requires the baseline pressure of 0 mmHg to be omitted. Figure 1 shows an example of the measured IAV and the estimated abdominal pressure–volume curve. The model is based on the assumption that there are parameters which defines the pressure–volume relation:

1. The maximum IAV, *V*_max_ rises, this shifts the horizontal asymptote up.
2. The abdominal cavity opens up at a lower insufflation pressure. The opening pressure,* p*_0_, shifts to the left.
3. The curvature of the pressure–volume relation changes, *λ*_exp._ A steeper increase in IAV takes place due to which the asymptotic *V*_max_ is reached at a lower IAP.

In a previous study [23], parameters *V*_max_,* p*_0_, and *λ*_exp_ were estimated using an empirical model:

$$IAV\left(p\right)={V}_{max}-\frac{{V}_{max}}{{e}^{{\lambda }_{exp}\bullet (IAP-{p}_{0})}}$$

This model was fitted to the pressure–volume curves of each insufflation run using Matlab (R2023b, Mathworks, Natick, Massachusetts, U.S.). Each parameter was tested using a linear mixed model with NMB group and insufflation repetition as fixed effects and subject number as random effects. The analysis was performed in R Studio (2022.07.2, R Foundation for Statistical Computing, Vienna, Austria). Linear mixed models were estimated for each of the three outcome parameters *V*_max_, *p*_0_, and *λ*_exp_. The independent variables (fixed effects) in the linear mixed models were NMB group (control, moderate, or complete) and insufflation run (REP). A random intercept of individual animals was included in the model to account for the within-subject correlations.

#### Cardiorespiratory effects

The cardiorespiratory effects investigated are PIP, MAP, HR, and CO. For each animal, these variables were repeatedly measured per insufflation run (REP) and pressure level at every insufflation step (STEP). For the analysis of the peak inspiratory pressure, an insufflation pressure of 0 mmHg was included in the analysis. To account for the structure of the data, a linear mixed effects model was developed with as independent variables NMB group, insufflation run and pressure level (as a categorical variable), and all two-way interactions between these three variables. A random intercept and random slope of the pressure level (as a continuous variable) were included for each animal and each insufflation run of each animal. The model was run separately for each cardiorespiratory effect. The results of the model are presented using the estimated marginal means, which are the predicted values of the outcome after adjusting for the effects of independent variables.

## Results

In total, 36 animals were investigated in this study. After five pilot experiments for refinement of the protocol, the insufflation measurement protocol was performed on 31 animals. One animal was excluded due to extensive pulmonary and cardiac adhesions. This animal was replaced to ensure equal groups of 10 for comparison. A total of 30 female Landrace pigs were included, their weights ranged from 18.5 to 24.1 kg (median 21.1 kg). Analysis of the CT scans and acquired physiological data resulted in 810 measures of IAV and cardiorespiratory parameters. During one experiment there was a malfunction of the data acquisition hub, leading to missing hemodynamic data. For this experiment, when available, the hemodynamic parameters were retrieved manually from the individual device logs and added to the results.

### Abdominal mechanics

**Fig. 2:**
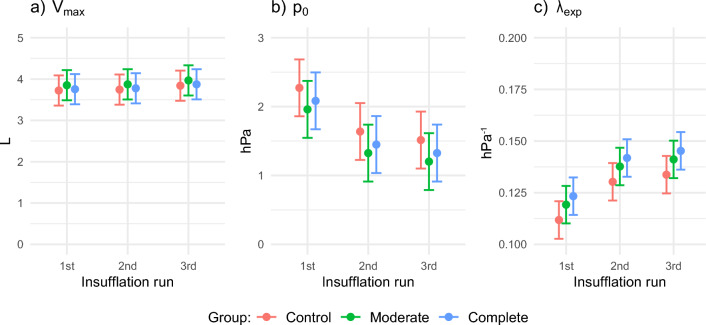
Abdominal pressure–volume curve, the effect of neuromuscular blockade and repeated insufflation, the estimated marginal means, and 95% confidence intervals based on the variation between animals. **a** The maximum intra-abdominal volume in L. **b** The opening pressure *p*_0_ in hPa. **c** The pressure expansion coefficient, *λ* in hPa^−1^

IAV ranged between 0 and 4.7 L, resulting in 90 estimations: three groups and three runs with ten animals per group. The effects of repeated insufflation on *V*_max_, *p*_0_, and *λ*_exp_ are consistent across different NMB conditions, with no significant influence from the specific type of neuromuscular blockade. After initial insufflation, the second and third insufflation showed a lower opening pressure, a higher maximum volume and an increased pressure expansion rate. The largest difference was observed between the initial insufflation and the 2nd insufflation. These gains diminished between the second and third insufflation. The results of the statistical analysis are added in Supplementary Tables 1, 2 and 3. A graphical summary of the results is given in Fig. 2, which shows the estimated marginal means of *V*_max_, *p*_0_ and *λ*_exp_. When compared to initial insufflation, in every group, *V*_max_ only increases significantly at the 2nd insufflation. In every group, the *p*_0_ is significantly lower after initial insufflation. The *λ*_exp_, is significantly higher after initial insufflation, indicating a higher abdominal compliance at the opening pressure.

#### Maximum IAV

In the control group, the mean *V*_max_ was 3.82 L with a confidence interval of 3.63–4.02 L. The effect of providing regular or complete NMB did not significantly alter *V*_max_ (*p* = 0.595). There was a significant increase in *V*_max_ between the initial insufflation and 2nd insufflation of 0.08 L with 95% CI 0.05–0.12 L (*p* < 0.001). There was no significant change in *V*_max_ between the 2nd and 3rd insufflation (*p* = 0.086). The estimated within-subject variance was 0.01, while the between-subject variance was 0.29.

#### Opening pressure

In the controls without NMB, the average opening pressure,* p*_0,_ was 1.64 hPa (1.23 mmHg), 95% CI 1.41–1.87 hPa. The effect of NMB was not significant, with a *p* value of 0.502 for the moderate NMB group and 0.371 for the complete NMB group. Repetition had a significant effect on the opening pressure. For the 2nd insufflation,* p*_0_ decreased by 0.54 hPa (0.41 mmHg), 95% CI −0.63 to −0.44 hPa (*p* < 0.001). For the 3rd insufflation,* p*_0_ increased by 0.21 hPa (0.16 mmHg), 95% CI 0.11–0.31 hPa (*p* < 0.001). The within-subject variance was 0.07, while the between-subject variance was 0.37.

#### Expansion coefficient

In the control group, the mean pressure expansion coefficient, *λ*_exp_, was 0.13 hPa^−1^ with 95% CI 0.13–0.14 hPa^−1^. When compared to the control group without NMB, the effect of NMB was not significant for moderate NMB (*p* = 0.56) and for complete NMB (*p* = 0.75). The effect of repeated insufflation was significant. There was an increase between the initial insufflation and 2nd insufflation, 0.016 hPa^−1^ with 95% CI 0.0118–0.0192 hPa^−1^ (*p* < 0.001). In the 3rd insufflation, λ_exp_ decreased, −0.0061 hPa^−1^ with 95% CI −0.0098 to −0.0024 hPa^−1^ (*p* = 0.001).

### Respiratory parameters

One measurement was excluded because the insufflation pressure setting was incorrect during the CT scan, 809 out of 810 data points were included. The respiratory pressure at the start of the 2nd and 3rd insufflation were higher (Supplementary Table 4). The required PIP initially reduces, indicative of improved respiratory compliance. At insufflation pressures exceeding the PEEP level of the mechanical ventilator (3.75 mmHg = 5 cmH_2_O), there is a linear relation between the increased insufflation pressure and the increase in PIP (*p* =  < 0.001).

The effect of repeated insufflation, the insufflation step, and its interaction significantly affects peak inspiratory pressure. Repeated insufflation increases peak inspiratory pressure significantly (*p* = 0.05). The insufflation pressure at each step significantly affects peak inspiratory pressure. The effect of NMB and its interactions with the insufflation step and repeated insufflation are not significant, with *p* = 0.54, *p* = 0.15, and *p* = 0.97, respectively. The interaction effect between NMB and repeated insufflation shows a non-significant effect on peak inspiratory pressure (*p* = 0.97). The interaction effect between repeated insufflation and insufflation step significantly affected peak inspiratory pressure (*p* < 0.001).

### Circulation

**Fig. 3:**
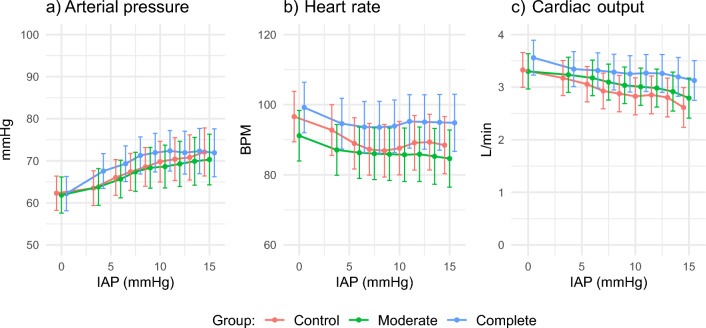
Circulation, the effect of insufflation pressure on mean arterial pressure, heart rate, and cardiac output. The graphs shows the estimated marginal means and 95% confidence interval based on the variation between animals. The columns sort the effect per group, the rows show the change of effect per insufflation run

One animal was excluded from the moderate NMB group due to missing data for the analysis of MAP. 783/810 observations were included for analysis. For HR and CO 809/810 observations were included. Figure 3 shows the effect of insufflation pressure on MAP, HR, and CO. Supplementary Tables 5, 6 and 7 include the corresponding ANOVA tables.

The changes in MAP, HR, and CO are strongly related to the applied insufflation pressure and the number of insufflations, rather than the depth of neuromuscular blockade. MAP increased with a rising insufflation pressure until reaching a plateau, with a more linear behavior observed with each subsequent run. The MAP at 15 mmHg was higher during the initial insufflation than during the 1st and 2nd repetitions. Differences between groups were notable, with the complete NMB group showing a steeper increase in MAP. The HR was higher in the complete NMB group, while the moderate NMB group showed similarities to the control group. The data showed that CO deteriorated with an increasing insufflation pressure.

## Discussion

This study used volumetric measurements to investigate the effects of changes in pressure, repeated insufflation and the administration of NMB on the size of the pneumoperitoneum and their effects on cardiorespiratory parameters. In our animal model for laparoscopy, repeated insufflation had a clear and positive effect on the size of the pneumoperitoneum, abdominal compliance, and ease of opening the abdominal cavity. Administering NMB had no significant effect on the mechanics of the abdominal cavity, even when administering a complete block.

### NMB effect on abdominal mechanics

Surgical stillness and voluntary breathing were not included as evaluation parameters; however, spasms were observed in the control group. Therefore, administering NMB is considered useful for mitigating these spasms**.** However, the results of this study indicate that NMB does not affect the maximum volume of the abdominal cavity. Taking into account Hill’s model for muscle mechanics [24], NMB does alter the control of muscle’s contractile elements, but even a paralyzed muscle retains its ‘passive’ elastic properties, the serial and parallel elements in Hill’s model, which are untouched by NMB. This study showed that regardless of the level of NMB, the passive muscle elasticity dominates the abdominal expansion behavior and the compliance of the abdominal cavity.

#### Abdominal compliance

This study in a homogeneous population showed a large variation in abdominal compliance. The variation in a more heterogeneous human population is expected to be even larger. This is in line with the study of Warle et al. [25]. Repeated insufflation greatly affected abdominal compliance, especially between the first and second insufflation. Repeated insufflation lowers* p*_0_ and increases compliance at this opening pressure. This proves that adequate workspace can be acquired at a lower pressure. To illustrate this, we selected a model that more accurately represents the pressure–volume (PV) curve expected in abdominal mechanics, as opposed to the conventional respiratory system-based models commonly described in literature [17]. These models are often derived from respiratory models and have an s-shape, but respiratory mechanics fundamentally differ from abdominal mechanics. Lungs will never fully close regardless of the pressure because of the adherence to the chest wall. Since the abdominal cavity does close, the compliance curve does not follow this s-shape. Selecting the alternative model was needed to be able to quantify the effects of NMB, REP and STEP. The model proved that repeated insufflation lowers threshold* p*_0_. In addition, the pressure expansion coefficient* λ*_exp_, reflects the abdominal compliance at this opening threshold. During the second insufflation, *λ*_exp_ went from 0.11 to 0.13 hPa^−1^, this is an 18% increase in compliance at the opening pressure.

#### Cardiorespiratory effects

At commonly applied insufflation pressures, IAP opposes lung ventilation, reducing lung compliance. In this study IAP’s exceeding 7.5 mmHg (10 hPa) decrease respiratory compliance, while lower pressures improve it. At 7.5 mmHg, there is no significant change from the baseline. This paradox may be explained by insufflation aiding exhalation when IAP is below the mean airway pressure. The cardiorespiratory effect of repeated insufflation remains unclear. During the second insufflation run the peak mechanical ventilation pressure was increased significantly by 1 cmH_2_O. During the third insufflation run, it was reduced significantly by 0.78 cmH_2_O. The interaction between the level of NMB and insufflation pressure showed a significant increase in peak ventilation pressures at 6, 7.5, 9, and 10 mmHg of insufflation. The relaxation of the diaphragm can explain the interaction between insufflation pressure and mechanical ventilation. The changes in MAP, HR, and CO are strongly related to the applied insufflation pressure and the number of insufflations rather than the depth of NMB. The body's cardiovascular response is significantly affected by how often (REP) and how much (STEP) insufflation pressure is applied; this emphasizes the importance of these variables in managing hemodynamics during minimal access surgery. The observed interaction effects highlight the interaction between these factors, necessitating careful consideration in clinical settings. Overall, taking cardiac output as the main circulatory parameter, increasing the insufflation pressure has a negative effect because MAP increases and heart rate decreases.

This study is supported by the use of a controlled porcine model, allowing for precise measurements and minimizing confounding variables. The model facilitated a comprehensive investigation, including CT scans with uniform mechanical ventilator settings. The oxygen and carbon dioxide levels were managed within normal values using the automated setup, minimizing their potential effect on the outcome of this study.

To maintain the same tidal volume, an increase in insufflation pressure required an increase in peak airway pressure of 50% of the insufflation pressure increment. Studies in humans showed ranges between 30 and 40% [17], this can be attributed to the different abdomen–thorax ratio in the porcine model, as pigs have relatively small lungs and a narrower thorax. Another difference between the porcine model and humans is the metabolic rate at which muscle relaxants are cleared: in the porcine model the metabolic rate is much higher [26]. However, in this study, the dosage of NMB was titrated to its effect on TOF/PTC and muscle relaxants were administered continuously to prevent this metabolic effect.

The study’s findings may be limited by the inherent differences between porcine and human physiology, the exclusive use of volume-controlled ventilation, and the highly controlled experimental conditions that may not fully reflect clinical variability. Future studies on insufflation should always correct for the effects of repeated insufflation and explore various ventilation modes. Especially since at lower insufflation pressures, the respiratory system compliance appears to benefit from the opposing insufflation pressure. To extrapolate these findings to different ventilation modes, it is essential to evaluate whether the effects occur primarily during inspiration or expiration. Hemodynamic changes during insufflation should be taken into account when refining the practice of laparoscopy, also incorporating the effects of NMB.

For the experimental protocol, continuous infusion of rocuronium was preferred over using boluses to maintain the desired level of NMB. This made it easier to titrate the NMB to the desired level and maintain stable relaxation. Throughout the *pilot* experiments, the authors noticed differences between the levels of NMB between upper and lower extremities. During insufflation, the upper extremities could be fully relaxed, while the lower extremities showed higher TOF/PTC levels. Throughout the *actual* experiments, the lower extremities were used for TOF/PTC testing. By infusing into both the internal jugular vein and femoral vein, the authors tried to mitigate these effects to the greatest extent possible. However, it could have affected the outcome of this study. Still, the results of this study show changes that are very relevant for clinical NMB management. Future studies should investigate whether a similar effect occurs in humans.

## Conclusion

In this study, in a porcine model, administering NMB, even to a level of complete neuromuscular blockade, did not alter the mechanics of the abdominal cavity and does not increase surgical workspace. The changes in MAP, HR, and CO were strongly related to the applied insufflation pressure and the number of insufflations rather than the depth of neuromuscular blockade. Repeated insufflation has a clear and positive effect on the pneumoperitoneum volume, abdominal compliance, and ease of opening the abdominal cavity and should be taken into account in future studies.

## Supplementary Information

Below is the link to the electronic supplementary material.

Supplementary file1 (DOCX 15 KB)

Supplementary file2 (DOCX 15 KB)

Supplementary file3 (DOCX 15 KB)

Supplementary file4 (DOCX 15 KB)

Supplementary file5 (DOCX 15 KB)

Supplementary file6 (DOCX 15 KB)

Supplementary file7 (DOCX 15 KB)
