# Supplementary material for: Surgical conditions in experimental laparoscopy: effects of pressure, neuromuscular blockade, and pre-stretching on workspace volume
Source: Surg Endosc. 2024 Oct 24;38(12):7426–34. doi: 10.1007/s00464-024-11338-0 (PMC11614944; doi:10.1007/s00464-024-11338-0)
Supplement: Supplementary file 1 — Supplementary file1 (DOCX 15 KB) [file 464_2024_11338_MOESM1_ESM.docx]

**Supplementary table 1** Results of the linear mixed model for the maximum volume, V_max_ (L)

| **Fixed effects** |  | |  |  |
| --- | --- | --- | --- | --- |
| **Predictors** | **Estimates** | | **Confidence interval** | **p**  **value** |
| **Control** | 3.82 | | 3.63 – 4.02 | **<0.001** |
| **Moderate** | 0.02 | | -0.31 – 0.36 | 0.893 |
| **Complete** | -0.09 | | -0.43 – 0.25 | 0.595 |
| **1st repetition** | 0.08 | | 0.05 – 0.12 | **<0.001** |
| **2nd repetition** | 0.03 | | -0.00 – 0.06 | 0.086 |
| **Random Effects** |  | |  |  |
| **σ^2^** | 0.01 | within-subject variance | | |
| **τ_00_Subject** | 0.29 | between-subject variance | | |
| **Marginal R^2^** | 0.019 | fixed factor variance | | |
| **Conditional R^2^** | 0.970 | total variance | | |
