# Supplementary material for: Surgical conditions in experimental laparoscopy: effects of pressure, neuromuscular blockade, and pre-stretching on workspace volume
Source: Surg Endosc. 2024 Oct 24;38(12):7426–34. doi: 10.1007/s00464-024-11338-0 (PMC11614944; doi:10.1007/s00464-024-11338-0)
Supplement: Supplementary file 2 — Supplementary file2 (DOCX 15 KB) [file 464_2024_11338_MOESM2_ESM.docx]

**Supplementary table 2** Results of the linear mixed model for the opening pressure, p_0_ (hPa).

| **Fixed effects** |  | |  |  |
| --- | --- | --- | --- | --- |
| **Predictors** | **Estimates** | | **Confidence interval** | **p**  **value** |
| **Control** | 1.64 | | 1.41 – 1.87 | **<0.001** |
| **Moderate** | -0.13 | | -0.53 – 0.26 | 0.502 |
| **Complete** | 0.18 | | -0.22 – 0.57 | 0.371 |
| **1st repetition** | -0.54 | | -0.63 – -0.44 | **<0.001** |
| **2nd repetition** | 0.21 | | 0.11 – 0.31 | **<0.001** |
| **Random Effects** |  | |  |  |
| **σ^2^** | 0.07 | within-subject variance | | |
| **τ_00_Subject** | 0.37 | between-subject variance | | |
| **Marginal R^2^** | 0.226 | fixed factor variance | | |
| **Conditional R^2^** | 0.873 | total variance | | |
