# Supplementary material for: Surgical conditions in experimental laparoscopy: effects of pressure, neuromuscular blockade, and pre-stretching on workspace volume
Source: Surg Endosc. 2024 Oct 24;38(12):7426–34. doi: 10.1007/s00464-024-11338-0 (PMC11614944; doi:10.1007/s00464-024-11338-0)
Supplement: Supplementary file 3 — Supplementary file3 (DOCX 15 KB) [file 464_2024_11338_MOESM3_ESM.docx]

**Supplementary table 3** Results of the linear mixed model for the expansion coefficient λ_exp_ (hPa^-1^).

| **Fixed effects** |  | |  |  |
| --- | --- | --- | --- | --- |
| **Predictors** | **Estimates** | | **Confidence interval** | **p**  **value** |
| **Control** | 0.13 | | 0.13 – 0.14 | **<0.001** |
| **Moderate** | 0.01 | | -0.00 – 0.02 | 0.056 |
| **Complete** | 0 | | -0.01 – 0.01 | 0.75 |
| **1st repetition** | 0.02 | | 0.01 – 0.02 | **<0.001** |
| **2nd repetition** | -0.01 | | -0.01 – -0.00 | **0.001** |
| **Random Effects** |  | |  |  |
| **σ^2^** | 0 | within-subject variance | | |
| **τ_00_Subject** | 0 | between-subject variance | | |
| **Marginal R^2^** | 0.3 | Fixed factor variance | | |
| **Conditional R^2^** | 0.713 | Total variance | | |
