# Supplementary material for: Surgical conditions in experimental laparoscopy: effects of pressure, neuromuscular blockade, and pre-stretching on workspace volume
Source: Surg Endosc. 2024 Oct 24;38(12):7426–34. doi: 10.1007/s00464-024-11338-0 (PMC11614944; doi:10.1007/s00464-024-11338-0)
Supplement: Supplementary file 4 — Supplementary file4 (DOCX 15 KB) [file 464_2024_11338_MOESM4_ESM.docx]

**Supplementary table 4** Respiratory mechanics, peak inspiratory pressure (cmH_2_O), the ANOVA table of the linear mixed model.

| *Peak Inspiratory Pressure (cmH_2_O)* | **Degrees  of freedom** | **Denominator  degrees  of freedom** | **F value** | **p**  **value** |
| --- | --- | --- | --- | --- |
| **NMB** | 2 | 32 | 0.63 | 0.54 |
| **REP** | 2 | 54 | 3.22 | **0.05** |
| **STEP** | 8 | 197 | 249.13 | **<0.001** |
| **NMB:STEP** | 16 | 197 | 1.40 | 0.15 |
| **NMB:REP** | 4 | 54 | 0.14 | 0.97 |
| **REP:STEP** | 16 | 645 | 7.21 | **<0.001** |

*^NMB^* ^Level of neuromuscular blockade,^ *^REP^* ^Insufflation repetition,^ *^STEP^* ^Insufflation step.^
