# Supplementary material for: Surgical conditions in experimental laparoscopy: effects of pressure, neuromuscular blockade, and pre-stretching on workspace volume
Source: Surg Endosc. 2024 Oct 24;38(12):7426–34. doi: 10.1007/s00464-024-11338-0 (PMC11614944; doi:10.1007/s00464-024-11338-0)
Supplement: Supplementary file 6 — Supplementary file6 (DOCX 15 KB) [file 464_2024_11338_MOESM6_ESM.docx]

**Supplementary table 6** Circulatory effects, heart rate (min^-1^), the ANOVA table of the linear mixed model.

| *Heart rate*  (min^-1^) | **Degrees  of freedom** | **Denominator  degrees  of freedom** | **F value** | **p**  **value** |
| --- | --- | --- | --- | --- |
| **NMB** | 2 | 31 | 1.42 | 0.26 |
| **REP** | 2 | 54 | 0.92 | 0.41 |
| **STEP** | 8 | 207 | 20.61 | **<0.001** |
| **NMB:STEP** | 16 | 207 | 2.03 | **0.01** |
| **NMB:REP** | 4 | 54 | 2.96 | **0.03** |
| **REP:STEP** | 16 | 651 | 0.94 | 0.52 |

*^NMB^* ^Level of neuromuscular blockade,^ *^REP^* ^Insufflation repetition,^ *^STEP^* ^Insufflation step.^
