# Supplementary material for: Surgical conditions in experimental laparoscopy: effects of pressure, neuromuscular blockade, and pre-stretching on workspace volume
Source: Surg Endosc. 2024 Oct 24;38(12):7426–34. doi: 10.1007/s00464-024-11338-0 (PMC11614944; doi:10.1007/s00464-024-11338-0)
Supplement: Supplementary file 7 — Supplementary file7 (DOCX 15 KB) [file 464_2024_11338_MOESM7_ESM.docx]

**Supplementary table 7** Circulatory effects, cardiac output (L/min), the ANOVA table of the linear mixed model.

| *Cardiac output*  *(L/min)* | **Degrees  of freedom** | **Denominator  degrees  of freedom** | **F value** | **p**  **value** |
| --- | --- | --- | --- | --- |
| **NMB** | 2 | 35 | 1.14 | 0.33 |
| **REP** | 2 | 52 | 2.45 | 0.10 |
| **STEP** | 8 | 241 | 12.36 | **<0.001** |
| **NMB:STEP** | 16 | 240 | 1.04 | 0.42 |
| **NMB:REP** | 4 | 54 | 1.43 | 0.24 |
| **REP:STEP** | 16 | 436 | 2.54 | **<0.001** |

*^NMB^* ^Level of neuromuscular blockade,^ *^REP^* ^Insufflation repetition,^ *^STEP^* ^Insufflation step.^
